# Using diffuse weighted image and apparent diffusion coefficient in MRI for diagnosis of posterior ischemic optic neuropathy in a young male: a case report and literature review

**DOI:** 10.1186/s12886-022-02379-x

**Published:** 2022-04-14

**Authors:** Tzu-Hsuan Yang, Muh-Chiou Lin

**Affiliations:** Department of Ophthalmology, Kaohsiung Veterans General Hospital, 386, Dazhong 1st Rd., Zuoying Dist, 81362 Kaohsiung City, Taiwan, Republic of China

**Keywords:** Posterior ischemic optic neuropathy, Inflammatory demyelinating optic neuritis, MRI diffusion-weighted image, Apparent diffusion coefficient

## Abstract

**Background:**

Posterior ischemic optic neuropathy (PION) is a rare cause of visual loss, especially in young patients who are more prone to inflammatory demyelinating optic neuritis (ON) compared to other types of optic neuropathy. The diagnosis of PION is usually a diagnosis of exclusion; however, the emergence of modern neuroimaging technique with diffuse-weighted image (DWI) and apparent diffusion coefficient (ADC) sequences in Magnetic Resonance Imaging (MRI) provides more evidence for accurate diagnosis and management.

**Case presentation:**

A 30-year-old man with a history of hypertension and chronic renal failure secondary to glomerulonephritis presented with sudden onset of blurred vision, dyschromatopsia, pain, and positive relative afferent pupillary defect (RAPD) in the left eye for 1 week. He was initially admitted for steroid pulse therapy and was further monitored due to suspicion of optic neuritis oculus sinister (OS). However, his brain MRI revealed a focal high hyperintensity signal at the left optic nerve on the T2 DWI series. The area was corresponded with the hypointensity area in the ADC series, which was a powerful clue for PION. We explained the poor visual prognosis of PION to the patient after finishing steroid pulse therapy and referred him to the Nephrology and Neurology department for hypertension control to prevent additional hypertension related complication.

**Conclusions:**

The diagnosis of PION is usually a diagnosis of exclusion; however, carefully interpreting the DWI and ADC sequence in MRI may give the clinician more evidence for the definite diagnosis and leads to proper management.

## Background

Disorders of the optic nerve represent a relatively common cause of visual loss. Among the numerous etiologies that result in optic neuropathy, inflammatory demyelinating ON and ischemic optic neuropathy (ION) are common among young and elderly patients, respectively [1, 2]. Although different optic neuropathies share similar clinical presentations, such as vision loss, dyschromatopsia, and positive relative afferent pupillary defect (RAPD), the prognosis is quite diverse. Recent advances in the neuroimaging have transformed how we diagnose and classify optic neuropathy and have provided more straightforward evidence for diagnosis. However, some of the useful sequences in MRI are still unfamiliar to ophthalmologists. Thus, we present a young male with acute vision loss who was initially suspected to have ON but his neuroimaging showed an unexpected result that required shifting the treatment plan.

## Case presentation

**Fig. 1:**
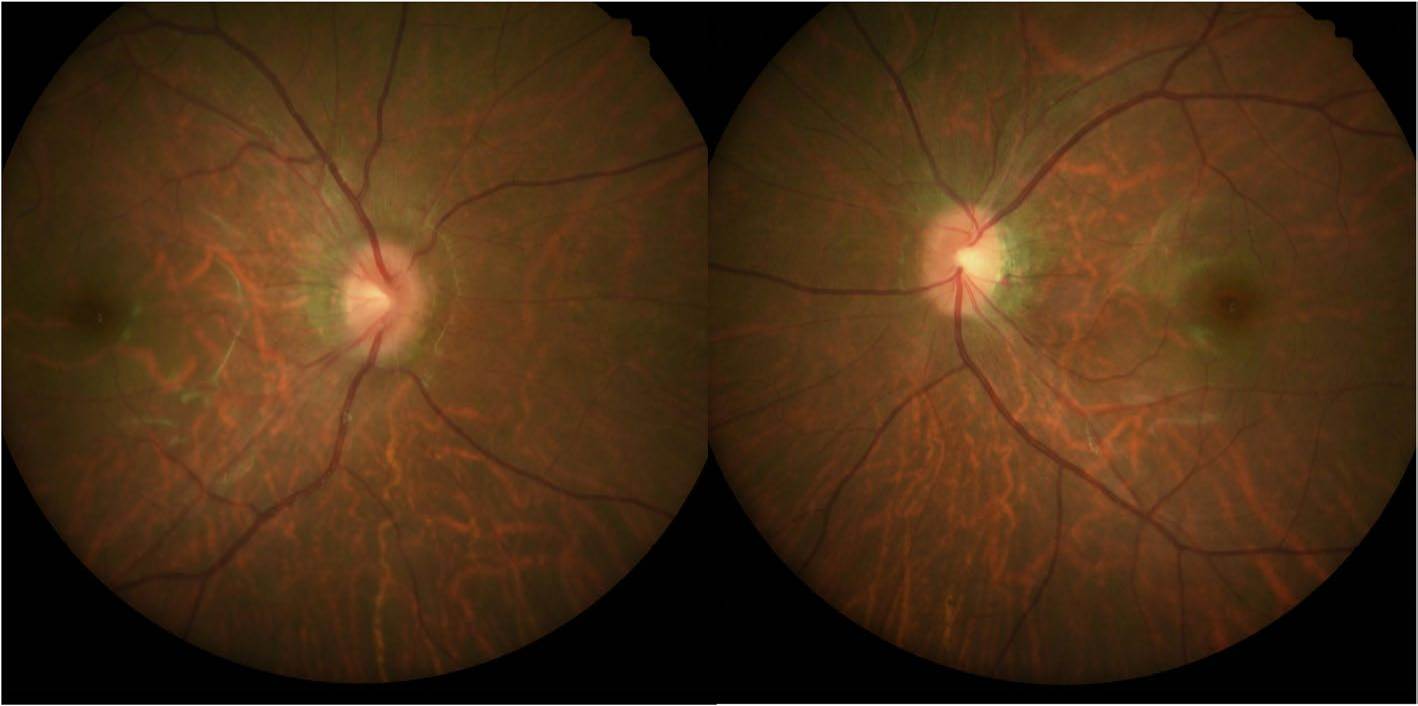
Fundus examination in both eyes

**Fig. 2:**
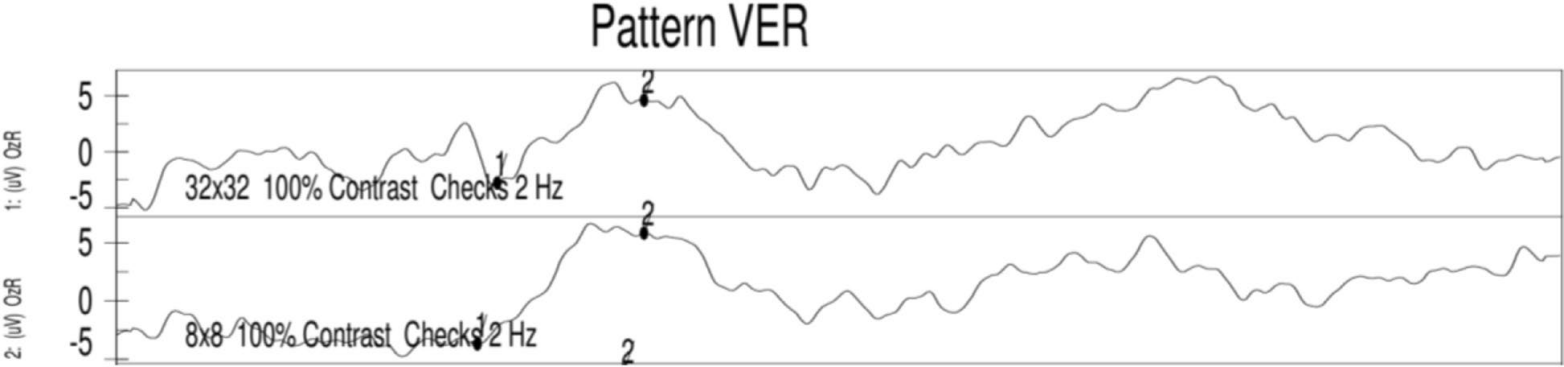
VEP showed delayed P1 wave with small amplitude in the left eye and normal response in the right eye

**Fig. 3:**
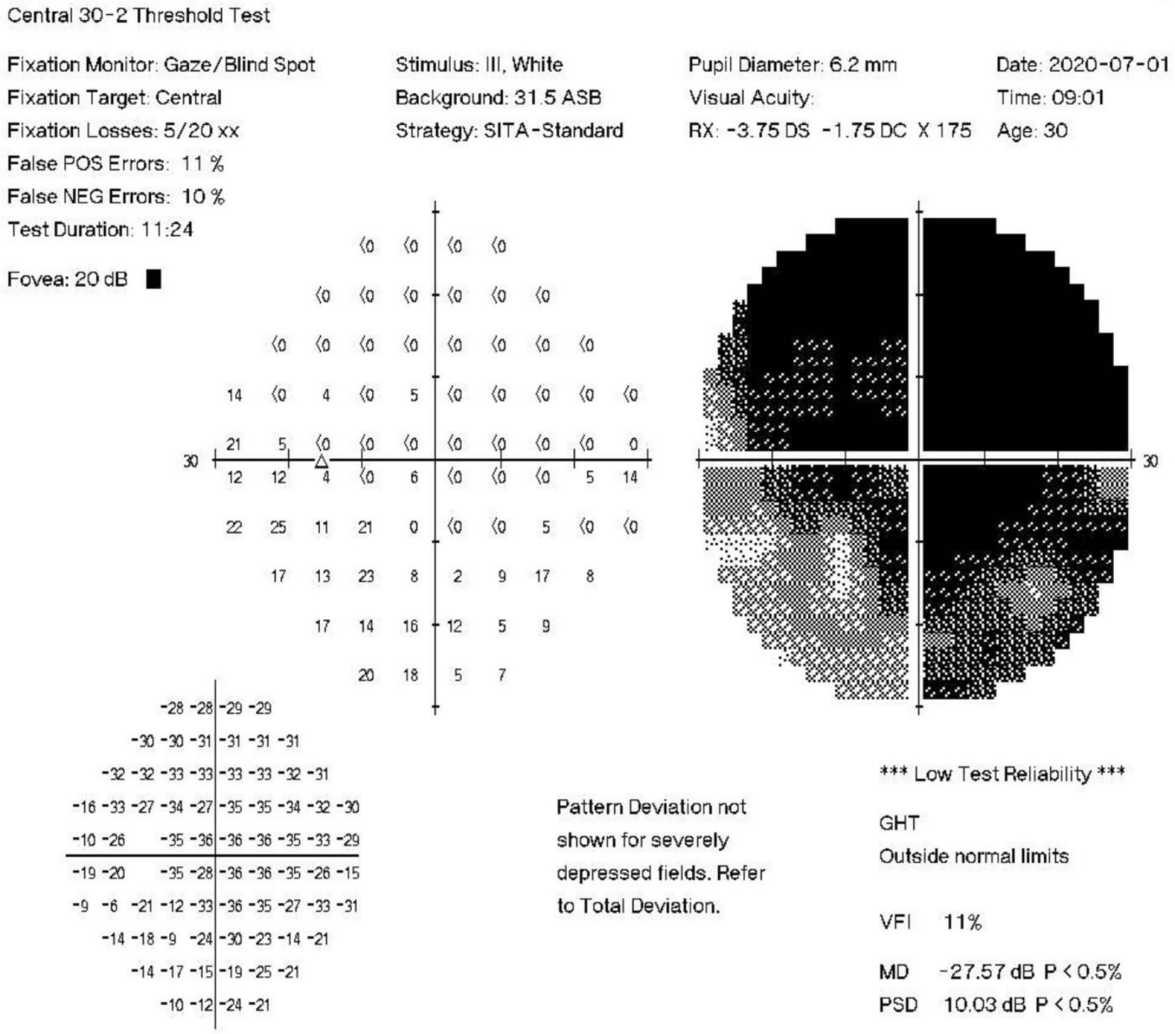
Humphrey visual field showed general depression in the left eye

A 30-year-old man presented with sudden onset of blurred vision and pain in the left eye 1 week before visiting our outpatient clinic. The patient had a history of chronic renal failure suspected as a consequence of childhood glomerulonephritis and complicated with hypertension. He was treated with oral furosemide, carvedilol, and nifedipine. Upon examination, he was high myopic with spherical refraction error of -8.50D in the right eye and − 6.75D in the left eye. His visual acuity was 20/20 in the right eye and counting fingers in the left eye. The Ishihara color test was normal in the right eye and 0 out of 21 plates in the left eye; Moreover, the RAPD in the left eye was strongly positive. The intraocular pressure was normal and no abnormality was seen in the anterior segment and fundus examination in both eyes (Fig. 1). Visual evoked potential (VEP) showed normal in the right eye and delayed response with small amplitude in the left eye (Fig. 2). Visual field showed normal in the right eye but general depression in the left eye (Fig. 3). Based on his clinical manifestations and features, he was admitted for further monitoring and started on methylprednisolone pulse therapy with 250 mg every 6 h for 3 days for the suspected demyelinating ON.

**Fig. 4:**
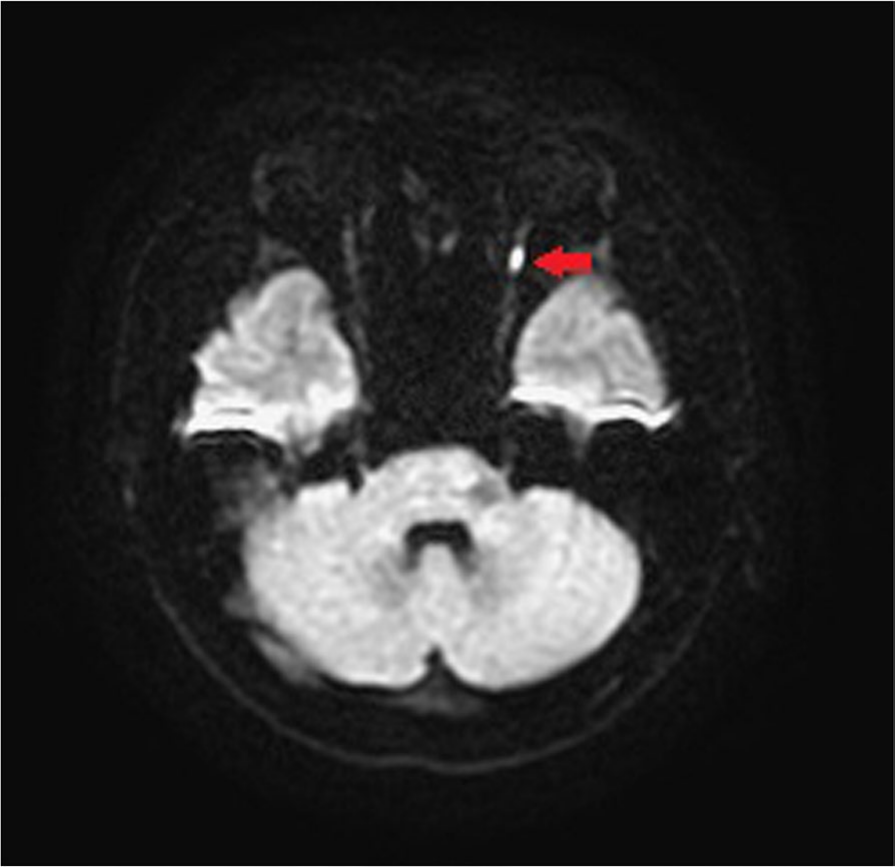
T2WI DWI showed focal high intensity signal at the left optic nerve. (Red arrow)

**Fig. 5:**
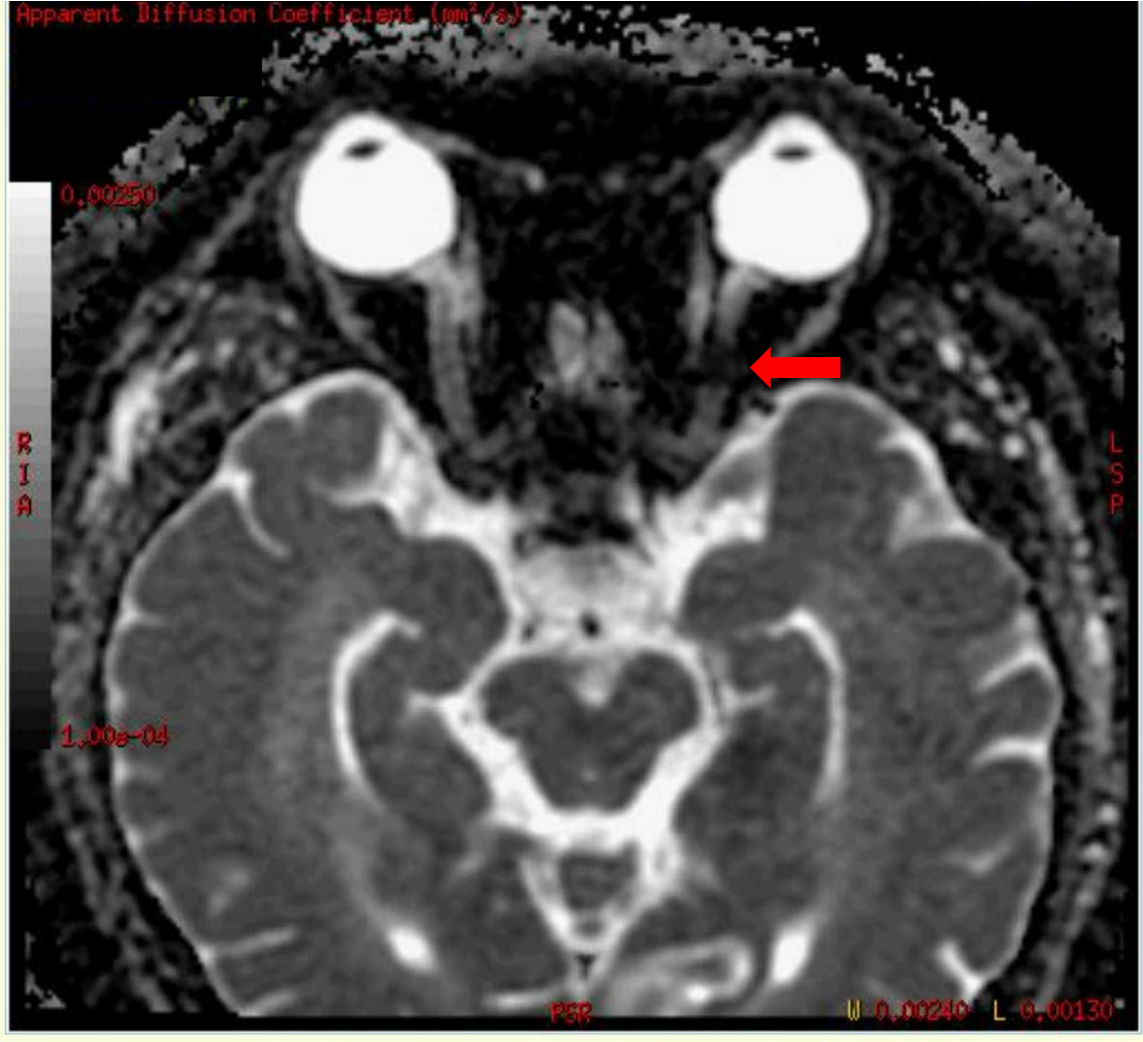
The hypointensity area shown on the ADC correlates to the hyperintensity area of DWI (Red arrow)

**Fig. 6:**
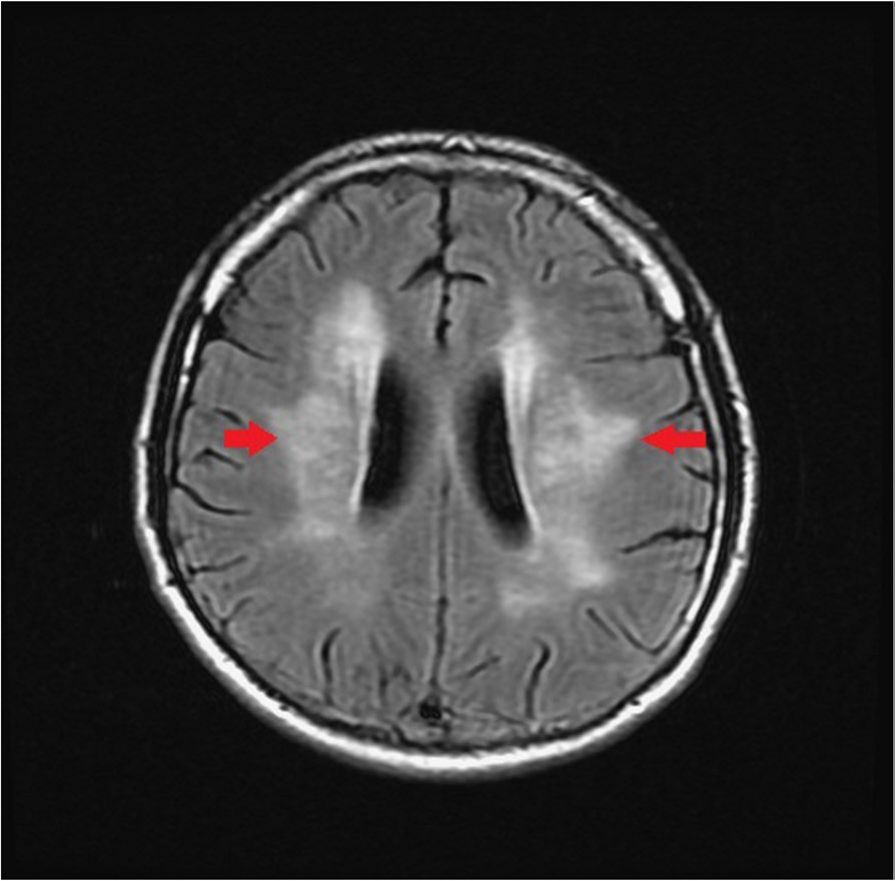
Multiple patches of high signal intensities involving bilateral periventricular white matter on T2-weighted and FLAIR image (Red arrow)

**Fig. 7:**
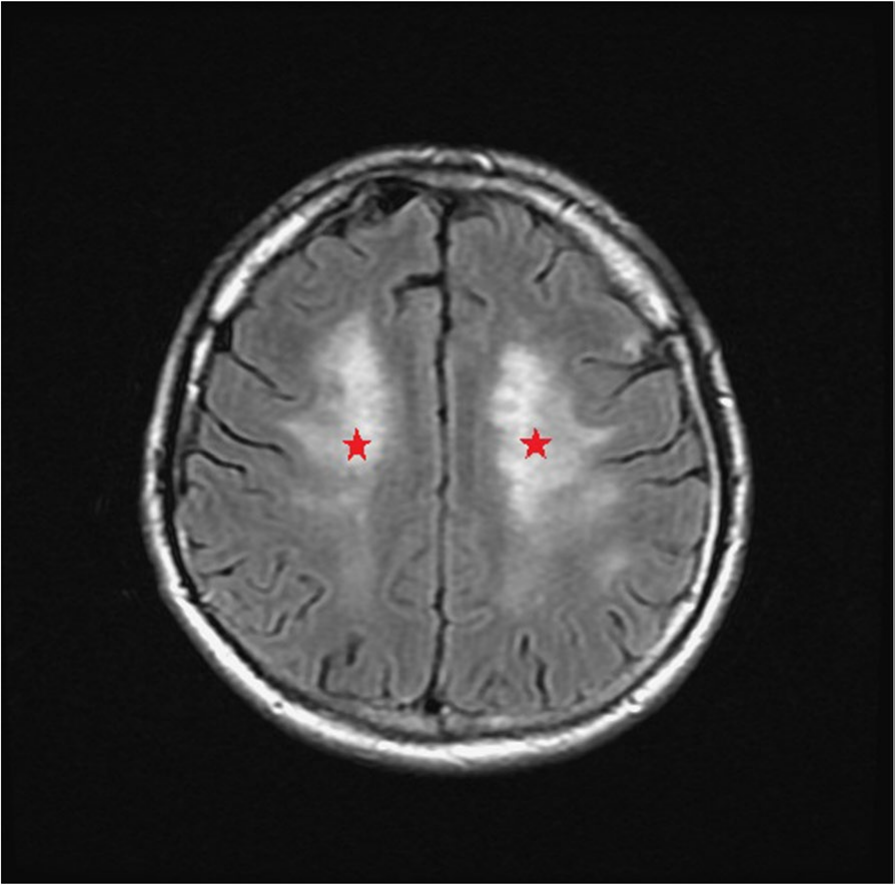
Multiple patches of high signal intensities involving bilateral periventricular white matter on T2-weighted and FLAIR image (Red stars.)

**Fig. 8:**
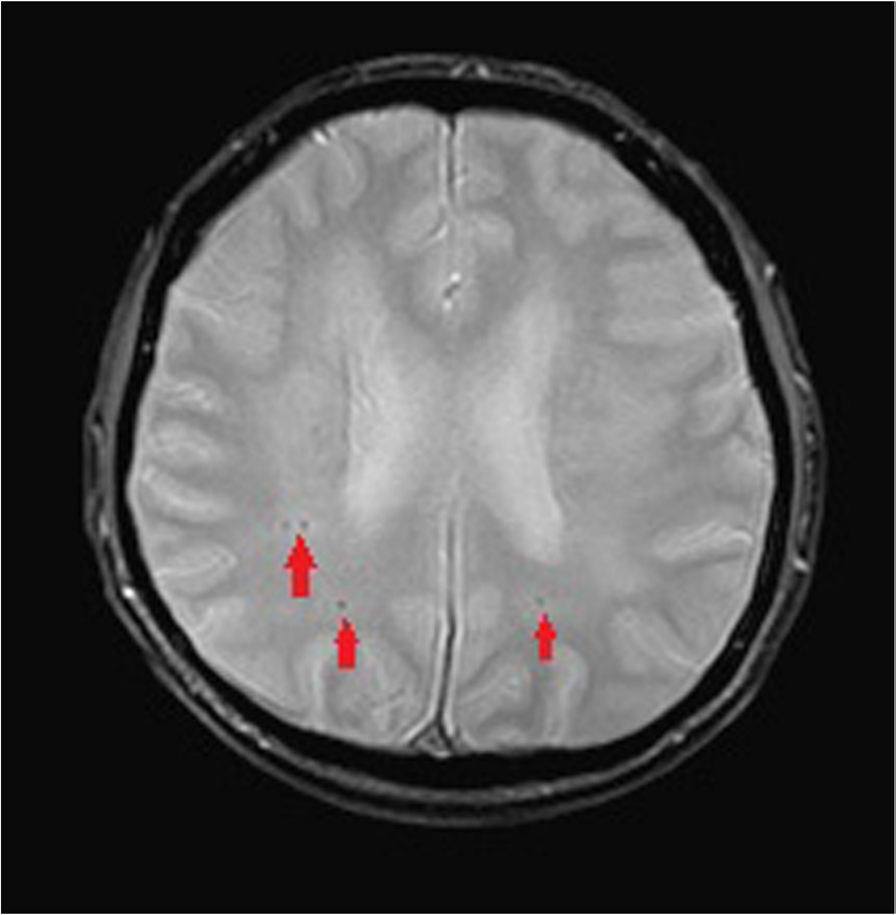
The hypointensity on the Axi MPGR (MPGR = multiplanar gradient-recalled) series on brain MRI indicates multiple small old ICH in multiple areas, leading to a strong suspicion of hypertensive microangiopathy ICH. (Red arrows)

**Fig. 9:**
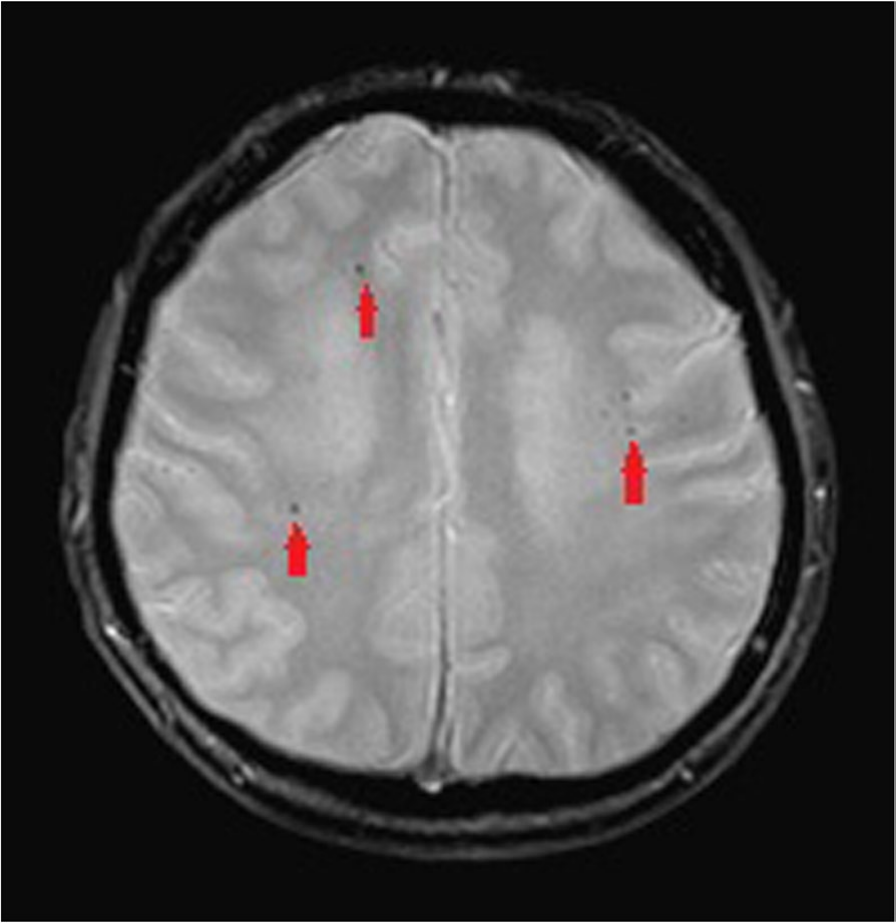
The hypointensity on the Axi MPGR (MPGR = multiplanar gradient-recalled) series on brain MRI indicates multiple small old ICH in multiple areas, leading to a strong suspicion of hypertensive microangiopathy ICH. (Red arrows)

During admission, refractory hypertension with systolic blood pressure up to 190 mmHg and diastolic blood pressure up to 127 mmHg were noted even with multiple anti-hypertensive medications. Lab data showed poor renal function with serum creatinine level up of 3.27 mg/dL, anemia, and hyperlipidemia with cholesterol level of 259 mg/dL and LDL level of 170 mg/dL. Fluorescence angiogram was arranged but was cancelled due to poor renal function. Brain MRI revealed a focal high hyperintensity signal at the left optic nerve on the T2 DWI series (Fig. 4). Furthermore, the hyperintensity area in the DWI series showed hypointensity in the ADC series (Fig. 5). There were multiple patches of high signal intensities involving bilateral periventricular white matter on T2-weighted and fluid-attenuated inversion recovery (FLAIR) images, which were suspected to be related to hypertensive subcortical arteriosclerotic encephalopathy (Figs. 6 and 7). Moreover, multiple small old intracranial hemorrhage (ICH) was also discovered in multiple areas including the pons, right basal ganglion, bilateral periventricular area, bilateral cerebellar, and cerebral hemisphere, thereby leading to a strong suspicion of hypertensive microangiopathy ICH (Figs. 8 and 9). According to the findings, PION was highly suspected related to his poor control hypertension secondary to chronic kidney disease. Therefore, the patient was referred to our neurology and nephrology specialists for further management of his systemic disease and blood pressure. At 6 month follow up, left eye visual acuity improved to 20/400, the color vision improved to 2 out of 21 Ishihara plates and the optic disc showed pallor. His renal function improved with serum creatinine level reduced to 2.29 mg/dL after strict diet and blood pressure control for 1 year.

## Discussion and conclusion

Acute ON due to inflammatory demyelination process is the most common optic neuropathy affecting young adults. It is characterized by acute unilateral vision loss, positive RAPD and pain with eye movement. Patients with typical ON are usually expected to have a significant recovery of visual acuity in weeks to months after their initial symptoms [1]. Although its clinical manifestation is similar with ON, ION usually occurs in the elderly, with an annual incidence estimated at 2.3 to 10.2 cases per 100,000 persons 50 years of age or older, and it seldom occurs in young people [3]. ION is the result of vascular insufficiency instead of inflammation, which is classified into anterior and posterior ION depending on the segment of optic nerve that is affected. Anterior ION involves optic nerve head and presents with disc edema while PION involves retrobulbar optic nerve and shows normal optic disc [4]. PION is relatively uncommon compared with its counterpart. Hayreh SS analyzed 1400 patients and found the relative frequencies of AION and PION were 96% and 4% respectively [2, 3]. The etiology of PION can be classified into three main categories: arteritic PION due to giant cell arteritis, non-arteritic PION usually associated with vascular risk factors, and perioperative PION in patients who have undergone prolonged surgery [2, 4, 5]. Non-arteritic PION is a multifactorial disease, Hayreh SS retrospectively reviewed 53 eyes with PION of 42 patients and found that the non-arteritic PION group has a higher prevalence of hypertension(*p* = 0.022), diabetes (*p* = 0.014), ischemic heart disease (*p* = 0.026), cerebrovascular disorder (*p* = 0.006), carotid artery and peripheral vascular disease (*p* < 0.0001), migraine (*p* = 0.039), and gastrointestinal ulcer (*p* = 0.011) than the age-matched control population in the US Caucasian population [4, 5]. These disease may contribute risk factors for the development of non-arteritic PION.

The diagnosis of PION is always a diagnosis of exclusion. The differential diagnosis of PION includes retrobulbar type ON, toxic optic neuropathy, compressive optic neuropathy and macular or retinal lesions. Therefore, thorough medical history and detailed ophthalmic exams including visual acuity, color vision test, anterior segment evaluation, fundus ophthalmoscopy, visual field, fluorescent angiography and VEP should all be arranged. Central visual loss, alone or in combination with other types of visual field defects, is the most common visual field defect reported in the previous studies in PION [4, 5]. Images of orbit and brain should also be evaluated for differential diagnosis of compressive optic neuropathy and retrobulbar optic neuritis. Among the different etiologies for PION, arteritic PION can be diagnosed based on symptoms, signs and elevated erythrocyte sedimentation rate and C-reactive protein while surgical PION can be diagnosed according to the patient’s history; However, it is usually difficult to make the diagnosis with certainty for non-arteritic PION [5]. A high-quality MRI of the brain and orbit is crucial and helpful for definitive diagnosis as it can show significant differences between inflammatory demyelinating ON and PION, in addition to the interpretation on DWI and ADC sequences. DWI is a form of MRI based upon measuring the random Brownian motion of water molecules within a voxel of tissue, which is particularly useful in tumor characterization and cerebral ischemia [6]. However, a high signal on DWI images could be due to T2 shine through phenomena rather than restricted diffusion. To confirm true restricted diffusion, the DWI image should be compared to the ADC. ADC is used to quantify the extent of restricted diffusion of water molecules in ischemic lesions, which show reduction in acute stage, pseudonormalization in subacute stage, and permanent hyperintensity in chronic stage. An acute ischemic injury is recognized by an increased DWI signal and decreased ADC value [6]. Theoretically, in PION, as in our case, there is ischemia of the pial capillary plexus, which supplies the posterior segment of optic nerve. Subsequent development of cytotoxic edema with restricted motion of water molecules produces an increased signal on DWI images and decreased ADC value. Conversely, inflammatory demyelinating ON, often shows an increased value in ADC and DWI sequence compared to PION due to axonal disruption, which may differentiate these two diseases at an acute stage [7].

The management of PION depends upon the type of PION. In arteritic PION caused by giant cell arteritis, high-dose systemic corticosteroid can prevent further vision loss [4]. In surgical PION, there is no treatment proved to be effective to recover or improve vision [5, 8]. As for non-arteritic PION, Hayreh conducted a randomized study and found that patients who received systemic corticosteroid treatment showed improvement in visual acuity (*p* = 0.023) and visual field (*p* = 0.03) compared with those without corticosteroid [2, 4, 5].

It is important to differentiate PION from inflammatory demyelinating ON because the prognosis is quite different. In inflammatory demyelinating ON, most patients have significant recovery of vision and nearly 85% return to baseline within 3 months of symptom onset even without treatment [1]. Sadda et al. retrospectively reviewed 98 eyes of PION and found that a significant proportion of patients with non-arteritic PION experienced visual improvement with three or more lines, while those with arteritic PION and perioperative PION show no change in vision even treated urgently and aggressively [9, 10].

To our knowledge, only few case reports of MRI findings of PION could be found in the literature and most were surgical PION [8]. Our case is the only case to be reported of non-arteritic PION showing restricted diffusion and is more unique for his young age.

In conclusion, appropriate interpretation of neuroimaging may aid in the correct diagnosis of PION and aid in the correct assessment of prognosis and providing adequate and accurate treatment for patients [11, 12].

## Data Availability

The datasets used and/or analyzed during the current study are available from the corresponding author on reasonable request.

## Abbreviations

PION: Posterior ischemic optic neuropathy
ON: Optic neuritis
DWI: Diffuse-weighted image
ADC: Apparent diffusion coefficient
MRI: Magnetic Resonance Imaging
OS: Oculus Sinister
ION: Ischemic optic neuropathy
VEP: Visual evoked potential
RAPD: Relative afferent pupillary defect
FLAIR: Fluid-attenuated inversion recovery
ICH: Intracranial hemorrhage
